# The Response of the Prostate to Circulating Cholesterol: Activating Transcription Factor 3 (ATF3) as a Prominent Node in a Cholesterol-Sensing Network

**DOI:** 10.1371/journal.pone.0039448

**Published:** 2012-07-02

**Authors:** Jayoung Kim, Dolores Di Vizio, Taek-Kyun Kim, Jonghwan Kim, Minjung Kim, Kristine Pelton, Steven K. Clinton, Tsonwin Hai, Daehee Hwang, Keith R. Solomon, Michael R. Freeman

**Affiliations:** 1 Division of Cancer Biology and Therapeutics, Departments of Surgery and Biomedical Sciences, Samuel Oschin Comprehensive Cancer Institute, Cedars-Sinai Medical Center, Los Angeles, California, United States of America; 2 The Urological Diseases Research Center, Children’s Hospital Boston, Boston, Massachusetts, United States of America; 3 Departments of Surgery and Biological Chemistry and Molecular Pharmacology, Harvard Medical School, Boston, Massachusetts, United States of America; 4 School of Interdisciplinary Bioscience and Bioengineering and Department of Chemical Engineering, Pohang University of Science and Technology, Pohang, Republic of Korea; 5 Molecular Cell and Developmental Biology, The University of Texas at Austin, Austin, Texas, United States of America; 6 Department of Molecular Oncology Department, H. Lee Moffitt Cancer Center, Tampa, Florida, United States of America; 7 Department of Orthopaedic Surgery, Children’s Hospital Boston, Harvard Medical School, Boston, Massachusetts, United States of America; 8 College of Medicine, The Ohio State University, Columbus, Ohio, United States of America; 9 Department of Molecular and Cellular Biochemistry, The Ohio State University, Columbus, Ohio, United States of America; Clermont Université, France

## Abstract

Elevated circulating cholesterol is a systemic risk factor for cardiovascular disease and metabolic syndrome, however the manner in which the normal prostate responds to variations in cholesterol levels is poorly understood. In this study we addressed the molecular and cellular effects of elevated and suppressed levels of circulating cholesterol on the normal prostate. Integrated bioinformatic analysis was performed using DNA microarray data from two experimental formats: (1) ventral prostate from male mice with chronically elevated circulating cholesterol and (2) human prostate cells exposed acutely to cholesterol depletion. A cholesterol-sensitive gene expression network was constructed from these data and the transcription factor ATF3 was identified as a prominent node in the network. Validation experiments confirmed that elevated cholesterol reduced ATF3 expression and enhanced proliferation of prostate cells, while cholesterol depletion increased ATF3 levels and inhibited proliferation. Cholesterol reduction in vivo alleviated dense lymphomononuclear infiltrates in the periprostatic adipose tissue, which were closely associated with nerve tracts and blood vessels. These findings open new perspectives on the role of cholesterol in prostate health, and provide a novel role for ATF3, and associated proteins within a large signaling network, as a cholesterol-sensing mechanism.

## Introduction

The human prostate is subject to a variety of pathologic conditions and syndromes that are not well understood in molecular terms. Most investigators have concluded that prostate health, particularly with respect to prostate cancer, is susceptible to lifestyle influences [1]. The association with lifestyle likely reflects a complex interplay between genetic, epigenetic, biochemical and metabolic processes. This is particularly evident in the US where sedentary habits and an obesogenic diet are now widespread. Accumulating evidence indicates that heart disease, diabetes and metabolic syndrome are associated with increased risk or severity of lower urinary tract symptoms (LUTS) [2]–[4].

Cholesterol, and structurally related lipids, are critical in membrane assembly and integrity. All eukaryotic cell membranes have cholesterol, ergosterol, or a phytosterol as a major membrane component. Cholesterol is one of the key regulators of membrane dynamics, a role tied to its tendency to facilitate the close packing of saturated acyl chains of membrane phospholipids, thereby stabilizing local membrane structure. Consequently, membrane compartmentalization into functional subdomains influenced by cholesterol concentration provides for post-translational control over important signal transduction pathways. The prostate synthesizes high levels of cholesterol, at similar rates to the liver, and the prostate accumulates cholesterol deposits with age [5]. Recent evidence from pre-clinical models has demonstrated a role for cholesterol in signal transduction in prostate cancer cells and tissues [6], [7], as well as in intraprostatic/intratumoral steroidogenesis [8] consistent with epidemiologic studies indicating that high circulating cholesterol promotes aggressive forms of the disease [9]–[12].

Normal tissues sense and respond to variations in circulating cholesterol. The adult prostate gradually loses the ability to regulate cholesterol levels at normal homeostatic levels, resulting in accumulation of excess intraprostatic cholesterol [5]. The degree or manner of the effect of this accumulation on prostate physiology is poorly understood. One community-based cohort study found a 4-fold increased risk of benign prostatic hyperplasia (BPH) among diabetic men with low density lipoprotein (LDL) cholesterol in the highest tertile in comparison to men in the lowest tertile [13]. A recent study from our group demonstrated that age-related prostate enlargement in the Syrian 87.20 hamster was dependent on the presence of cholesterol in the diet, and could be reversed with ezetimibe, a specific inhibitor of cholesterol absorption from the intestine [14]. However, our understanding of the effects of elevated cholesterol and hypocholesterolemic drugs on the prostate is limited. It is not known, for example, whether hypercholesterolemia elicits physiologic, metabolic, or biochemical changes in the prostate that predispose toward disease.

In the present study we attempted to uncover a cholesterol-sensitive signaling network in the normal prostate of the mouse and human prostate cells using a systems approach. Our findings indicate that the prostate responds to variations in circulating cholesterol levels by altering cholesterol tissue content, cell proliferation rate and gene expression. Evidence also suggests that hypocholesterolemia may suppress prostatic inflammation. Our findings provide the first broad look at the manner in which the normal prostate responds to changes in circulating cholesterol.

## Materials and Methods 

### Reagents

Heat-inactivated fetal bovine serum (FBS) and Lipofectamine 2000 were from Invitrogen (Carlsbad, CA). Protease inhibitor cocktail tablets were from Roche Diagnostics (Basel, Switzerland). The Micro BCA protein assay kit was obtained from Pierce (Rockford, IL). Coomassie Blue R-250 staining solution and destaining solution were from Bio-Rad (Hercules, CA). Small interfering RNAs (siRNAs) against ATF3 and NON-TARGET controls were from Dharmacon (Chicago, IL). 4’,6-diamidino-2-phenylindole (DAPI) was purchased from Vector Laboratories (Burlingame, CA). Antibodies against ATF3 and β-actin were purchased from Santa Cruz Biotechnology, Sigma-Aldrich (St. Louis, MO) and Cell Signaling Technology (Danvers, MA). Antibody against Cy3-conjugated AffiniPure goat anti-rabbit IgG, Fc fragment specific was obtained from Jackson Immuno Research (West Grove, PA). All other reagents were obtained from Sigma-Aldrich or Promega (Madison, WI).

### Animal Maintenance and Diet

Male SCID and C57BL/6 mice (5–6 wk old) were obtained from the Massachusetts General Hospital and Jackson Labs, respectively. All mice were maintained on a low fat, no cholesterol diet (LFNC) (Research Diets, New Brunswick, NJ diet # D12102), equivalent to normal mouse chow, but without lot to lot variation for 2 weeks to normalize cholesterol levels, after which mice were divided into individual diet groups. C57BL/6 mice were divided into 3 diet groups (n = 18/group): hypocholesterolemic (Hypo) (LFNC + ezetimibe–30mg/kg/day; Schering-Plough, New Brunswick, NJ) added to the food), normocholesterolemic (Normo) (LFNC diet) or hypercholesterolemic (Hyper) (high fat, high cholesterol (HFHC) diet) (Research Diets, diet # D12108). SCID mice were grouped into 2 diet groups; Hyper and Normo. Animals were maintained on the respective diets for 4 months. Animals were housed in compliance with Children’s Hospital Boston's animal care and use policies. All procedures were approved by IACUC under protocols A07-06-084 and 07-08-1503. Terminal bleeds were taken for liver function testing and serology by cardiac puncture. Prostates were removed and divided into individual anatomic lobes, i.e. ventral (VP), dorsal lateral (DLP) and anterior (AP) under a dissecting microscope, and either placed in OCT solution (Tissue-Tek, Torrance, CA) or in formalin for paraffin embedding [15].

### Immunohistochemistry and Immunofluorescence Cell Staining

4-µm cryostat-obtained tissue sections were mounted on Superfrost/Plus microscope slides and kept at –80°C before use. Frozen sections were kept at RT for 1 min, followed by 3X washes in PBS. Fixation was done with Permeabilization Solution (BD Bioscience, San Diego, CA) for 15 min. After 1 h blocking with 3% BSA/PBS solution, slides were incubated for 12 h at 4°C with specific antibodies, followed by incubation with HRP-conjugated secondary IgG for 30 min at RT. After washing with PBS (3X), 3-3’ diaminobenzidine (DAB) substrate chromogen solution (Envision Plus Kit, Dako Corp) was applied. The reaction was monitored by microscopy and was terminated when properly developed. Nuclei were counterstained with Meyer’s hematoxylin and slides were analyzed using an Axioplan 2 microscope (Carl Zeiss MicroImaging, Inc. Thornwood, NY). Paraffin slides were dried at 60°C for 2 h before immunohistochemical staining. After deparaffinization, rehydration and depletion of endogenous peroxidase activity as described [15], tissue was stained with specific antibodies against Ki-67 (1∶3000, Abcam, Cambridge, MA) and ATF3 (1∶100, Santa Cruz Biotechnology, Santa Cruz, CA). For immunofluorescence staining, slides were incubated with antibodies, followed by incubation with Cy3, or FITC-conjugated secondary Abs (Molecular Probes) as described [16], [17].

### Proliferation Analysis by Ki67 Staining

Cellular proliferation was analyzed by immunohistochemical staining of formalin-fixed tissues using a rabbit polyclonal anti-Ki67 antibody. Ten randomly chosen sections from 5 animals per group were used for analysis. The number of Ki67-labeled nuclei in total 5000 hematoxylin-stained prostate epithelial cells was quantified using an Axioplan 2 microscope.

### Cell Culture and Transfection

LNCaP human prostate tumor cells were purchased from American Type Culture Collection (ATCC, Manassas, VA) and maintained in RPMI1640 media (Invitrogen, Carlsbad, CA) supplemented with 10% FBS and 1% Penicillin/Streptomycin at 37°C under 5% CO_2_. The hTERT-immortalized normal prostate epithelial cells (PrEC) cells were generously provided by Dr. William Hahn (Dana-Farber Cancer Institute, Boston, MA) [18] and maintained in PrEGM medium. Cells were grown to ∼80% confluence, at which time they were transiently transfected with small interfering RNAs (siRNAs) or gene expressing constructs. To silence ATF3 expression, cells were transiently transfected with ATF3 ON-TARGETplus SMARTpool siRNAs as well as 4 independent ATF3 siRNAs duplexes (Dharmacon Inc., Chicago, IL) using Nucleofector (Amaxa, Walkersville, MD). For ATF3 overexpression, an ATF3 construct (or empty vector control) was used for transient transfection of LNCaP cells.

### Measurement of Cholesterol Level

Tissues or cells were finely minced in PBS on ice, membranes were isolated as described [19], and cholesterol levels were determined after lipid extraction using the Infinity Cholesterol Liquid Stable Reagent (Thermo Electron Corp., Waltham, MA).

### Flow Cytometry

LNCaP cells were incubated in control medium or cholesterol-depletion medium for 18 h.

After trypsinization, cells were resuspended in PBS containing 2% FBS, and fixed in absolute ethanol at 4°C for 1 h. Cells were resuspended in 50 µg/mL propidium iodide staining solution and subjected to flow cytometry using a FACSCalibur flow cytometer (BD Biosciences). The apoptotic population of cells (sub-G_0_/G_1_) was calculated by using CellQuest Pro (BD Biosciences).

### RNA Extraction

Total RNA was purified from prostate lobes or cultured cells using a Qiagen RNEasy tissue extraction kit (Qiagen Inc., Valencia, California). RNA concentration was measured using a Nanodrop ND-1000 spectrophotometer (Thermo Scientific, Willmington, DE).

### Microarrays and Data Analysis

Reverse transcription of total RNA and subsequent steps for sample probe preparation, microarray hybridization, washing and scanning of microarrays were performed by following a standard Affymetrix protocol at the Dana-Farber Cancer Institute microarray core facility. Oligo-based Mouse Genome 430A 2.0 Arrays (Affymetrix, for mouse microarray) or Human Genome U133A 2.0 Array (NuGEN V2, for LNCaP microarray) were used, respectively. For the mouse array experiment, two biological repeats were performed, while for the human array experiment, single samples were performed across a time course. Data normalization and summarization of expression values for each probe were calculated using the gcRMA method. We used the annotations of probesets that were provided by the Affymetrix website. All raw microarray data discussed here are deposited and available at Gene Expression Omnibus (http://www.ncbi.nlm.nih.gov/geo/) under the accession number GSE25500.

For the LNCaP data set, we then identified the differentially expressed genes (DEGs) as the genes with absolute log2-fold-changes with respect to CDM/time 0>0.585 (i.e. 1.5 fold). For the Hyper diet data, we used the following integrative hypothesis testing method: 1) we performed two-tailed T-test and log2-median-ratio tests; 2) false discovery rates (FDRs) were computed using all possible randomization experiments for the individual statistical tests using Storey’s method [20]; 3) the FDRs from the individual statistical tests were combined to result in the overall FDRs using Stouffer’s method [21]. The DEGs were selected as the ones whose overall FDRs were <0.05 and also absolute values fold changes >0.585. To integrate differential expression patterns of the DEGs in mouse and human, we used the ontology information obtained from the Mouse Genome Informatics (MGI) database (ver. 4.4). We searched for Gene Ontology Biological Processes (GOBPs) and known pathways that were statistically enriched by the DEGs using the DAVID software [22].

### Network Modeling

We constructed a hypothetical network model using the 265 DEGs altered in both the hypercholesterolemic diet and CDM conditions. Information on protein-protein and protein-DNA interactions was gathered from the Kyoto Encyclopedia of Genes and Genomes (KEGG) and NCBI databases. We generated an initial network using the first neighbors of the 265 DEGs based on the interaction data. The initial network was then reduced to generate a subnetwork by removing the first neighbors of the DEGs that do not contribute to connecting DEG nodes. Finally, the nodes were grouped based on functional similarities based on GOBPs. To identify key central regulators among the DEGs, we computed ‘degree centrality’ of each regulator in the network [23]. A DEG regulator with high degree of centrality is considered a critical component that can regulate biological processes in the network [24].

**Figure 1:**
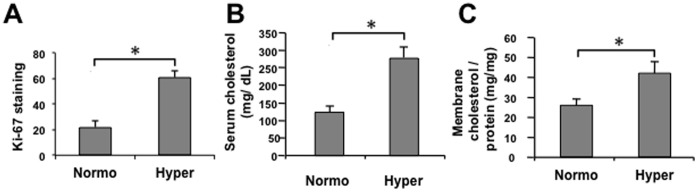
Effect of dietary cholesterol on circulating and prostatic tissue cholesterol levels *in vivo*. Male SCID mice were fed for 4 months (m) either a Hyper or a Normo diet, and circulating as well as prostatic tissue cholesterol levels determined. (**A**) Hyper diet enhances prostatic proliferation. 10 randomly selected sections per group were used for analysis with proliferating cells determined by Ki-67 staining. Ki-67 positivity is shown as average ± SD (n = 10/group) of positive cells in a total of 5000 prostate epithelial cells. (**B**) **Circulating cholesterol levels.** Serum cholesterol levels were determined and are plotted as cholesterol (mg/dL) vs. diet group ± SD (n = 15/group) (**C**) **Cholesterol levels of prostate membrane.** Cholesterol was extracted from membrane fractions prepared from prostate tissue and cholesterol levels determined by Infinity assay. Data are presented as cholesterol (mg/mg tissue) vs. group ± SD (n = 3/group). *p<0.05 (Student’s t-test).

### Reverse Transcription and PCR

Primers for RT-PCR were designed using Mac Vector and were ordered from Integrated DNA Technologies (Coralville, IA). The cDNA amplification was performed using Invitrogen Platinum Blue PCR Supermix (Carlsbad, CA).

### Cell Lysis and Western Blot Analysis

Tissue or cells were finely minced in PBS on ice and whole cell lysates were prepared in SDS-containing lysis buffer (62.5 mM Tris pH 7.4, 10% glycerol, 1% SDS and protease inhibitor cocktail) or in NP-40-containing lysis buffer (1% Nonidet P-40, 50 mM Tris pH 7.4, 10 mM NaCl, 1 mM NaF, 5 mM MgCl_2_, 0.1 mM EDTA, 1 mM PMSF and protease inhibitor cocktail). After centrifugation at 12,000×g for 15 min to remove debris, protein concentration was determined by microBCA (Pierce/Thermo Scientific). Equal amounts of the lysates (20 µg) were subjected to SDS-PAGE and transferred onto nitrocellulose membranes for western blot analysis. Blotting signals were normalized by reprobing blots with anti-β-actin antibody, followed by densitometry for quantification.

### Promoter Luciferase Assay

Luciferase reporter constructs with ATF3 promoter, wild type cyclin D1 promoter, or ATF3 binding mutant cyclin D1 promoter constructs were used for transient transfection of LNCaP or PrEC. Luciferase activity was measured in cell lysates using a luciferase assay kit (Promega, Madison, WI). Total protein was used for normalization. All experiments were carried out in triplicate and repeated 3 times using different preparations of plasmids.

**Figure 2:**
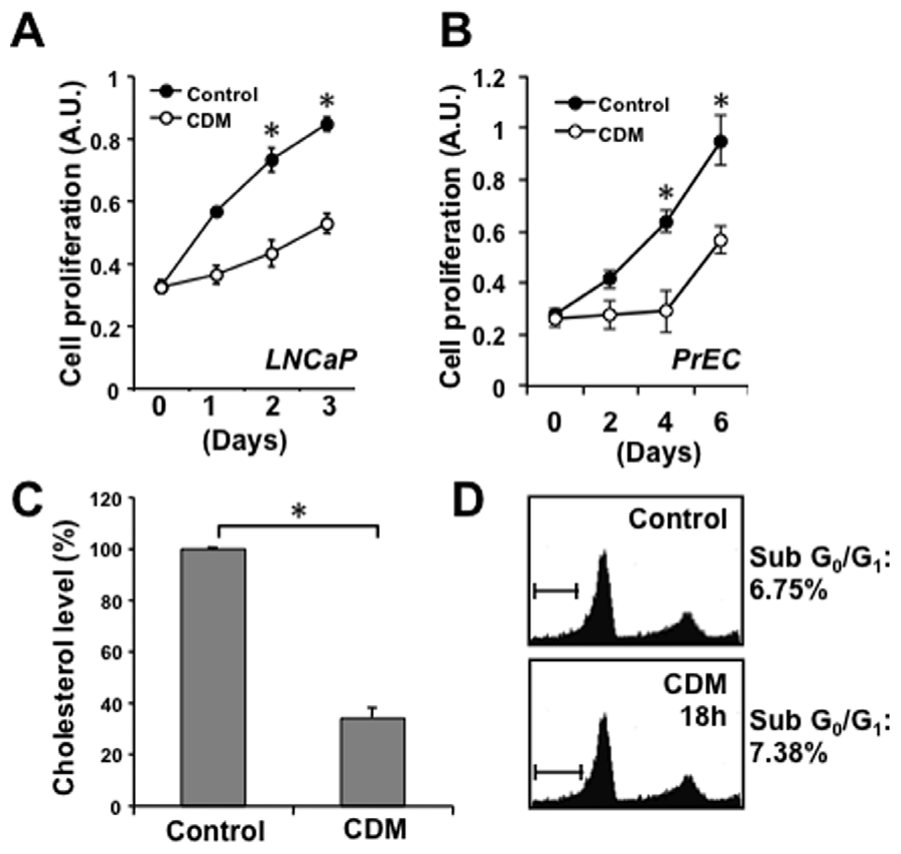
Cholesterol depletion reduces cellular cholesterol levels and inhibits proliferation without inducing apoptosis. (**A–B**) Cholesterol depletion reduces cellular proliferation of LNCaP (A) and PrEC cells (B). Cell proliferation was determined at the indicated times by crystal violet staining. Data are plotted as cell proliferation (A.U., absorption units) vs. time (days) ± SD (n = 5). (**C**) **Incubation in cholesterol-depleted media (CDM) reduces cellular cholesterol levels.** LNCaP cells were incubated in control media (RPMI 10% FBS) or CDM for 18 h. Cholesterol level data are presented as percent cholesterol vs. treatment ± SD (n = 3). (**D**) **Cholesterol depletion does not induce apoptosis.** LNCaP cells were treated in control media or CDM for 18 h and were analyzed for levels of apoptosis by flow cytometry. Cell populations at Sub-G_0_/G_1_ are apoptotic. *p<0.05 (Student’s t-test).

### Cell Proliferation Assay

LNCaP or PrEC were plated onto 6-well culture plates at a density of 1×10^3^ cells per well in standard growth medium. The following day cells were serum-starved for 16 h followed by treatment with the indicated medium: SF (serum free), CDM (cholesterol-depleted medium) or control medium. Cell proliferation rate was determined by crystal violet staining. Briefly, cells were stained with crystal violet solution and quantified by dissolving stained cells in 10% acetic acid solution. Colorimetric analysis was performed by measuring absorbance at 570nm.

### Assessment of Inflammation

The inflammatory infiltrate detected in the peri-prostatic adipose tissue of H&E stained slides was subjected to semi-quantitative scoring as follows: 0 (absent; no infiltrate), 1 (mild; focal, scattered monocytoid cells), 2 (moderate; monocytoid cells organized in small lymphoid follicles), and 3 (severe; monocytoid cells organized in large lymphoid follicles).

### Statistical Analysis

P-values were calculated using unpaired Student’s t-tests or two-way ANOVA for simple comparisons. P<0.05 is considered statistically significant.

**Figure 3:**
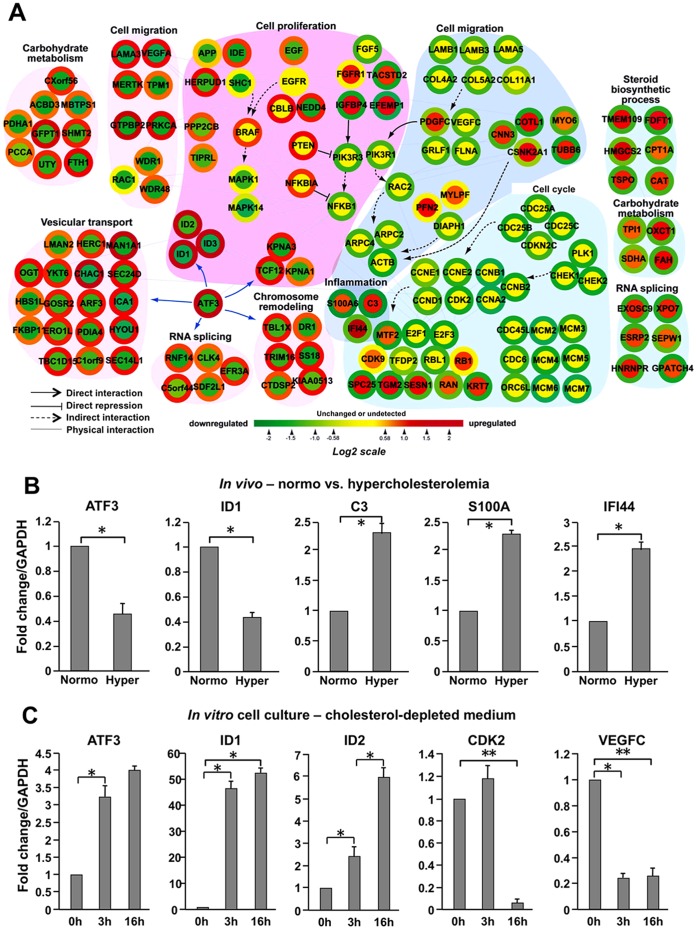
Network modeling of the cholesterol-responsive genes. (**A**) **A provisional network was generated from integration of two microarray data sets.** Node color represents increases (red), no significant changes (yellow), and decreases (green) in gene expression in murine prostate tissue after cholesterol alteration as ascertained by cDNA microarray. Changes in RNA expression levels of the corresponding nodes in LNCaP cells are shown as colored node boundaries (donut shape) and the color represents increases (red), no significant change (yellow), and decreases (green) in gene expression under CDM conditions compared to control. Arrows indicate direct activation, T-shaped lines direct repression, dashed arrows indirect activation, and lines physical interaction. (**B**) **Gene expression under Normo and Hyper conditions (** ***in vivo*** **).** To verify *in vivo* microarray data obtained from SCID experiments, mRNA levels of the indicated genes were determined. GAPDH expression was used to normalize gene expression. Error bars represent SD (n = 3). (**C**) **Gene expression under Control and Cholesterol-depleted conditions (** ***in vitro*** **).** LNCaP cells were incubated in CDM for 0, 3 or 16 h, and mRNA levels of the indicated genes were measured by RT-PCR analysis to validate cDNA microarray data. Error bars represent SD (n = 3). *p<0.05 (Student’s t-test).

## Results

### Molecular Responses to Variations in Cholesterol Level

In order to investigate the nature of the response of the normal mouse prostate to chronic changes in circulating cholesterol levels, we made 7–8 wk old male C57BL/6 and severe combined immunodeficient (SCID) mice (which lack T and B cells) hypercholesterolemic (the “Hyper” condition), and compared them with mice maintained at normal cholesterol levels (the “Normo” condition) for 4 months using an isocaloric diet procedure developed by our group [7]. Circulating testosterone/DHT, insulin levels, prostate size and prostate volume were not statistically different between the 2 groups (Hyper vs. Normo), and these diets did not cause weight gain/loss or liver dysfunction (data not shown). Histological examination of prostate tissue from immune intact C57BL/6 mice revealed extensive, dense inflammatory infiltrates in the periprostatic adipose tissue (Fig. S1), but not in prostate parenchyma, in the Normo group (not shown). In order to detect primarily parenchymal, instead of inflammatory, responses to changes in circulating cholesterol, we used SCID mice in subsequent RNA expression profiling experiments in which circulating cholesterol was manipulated.

**Figure 4:**
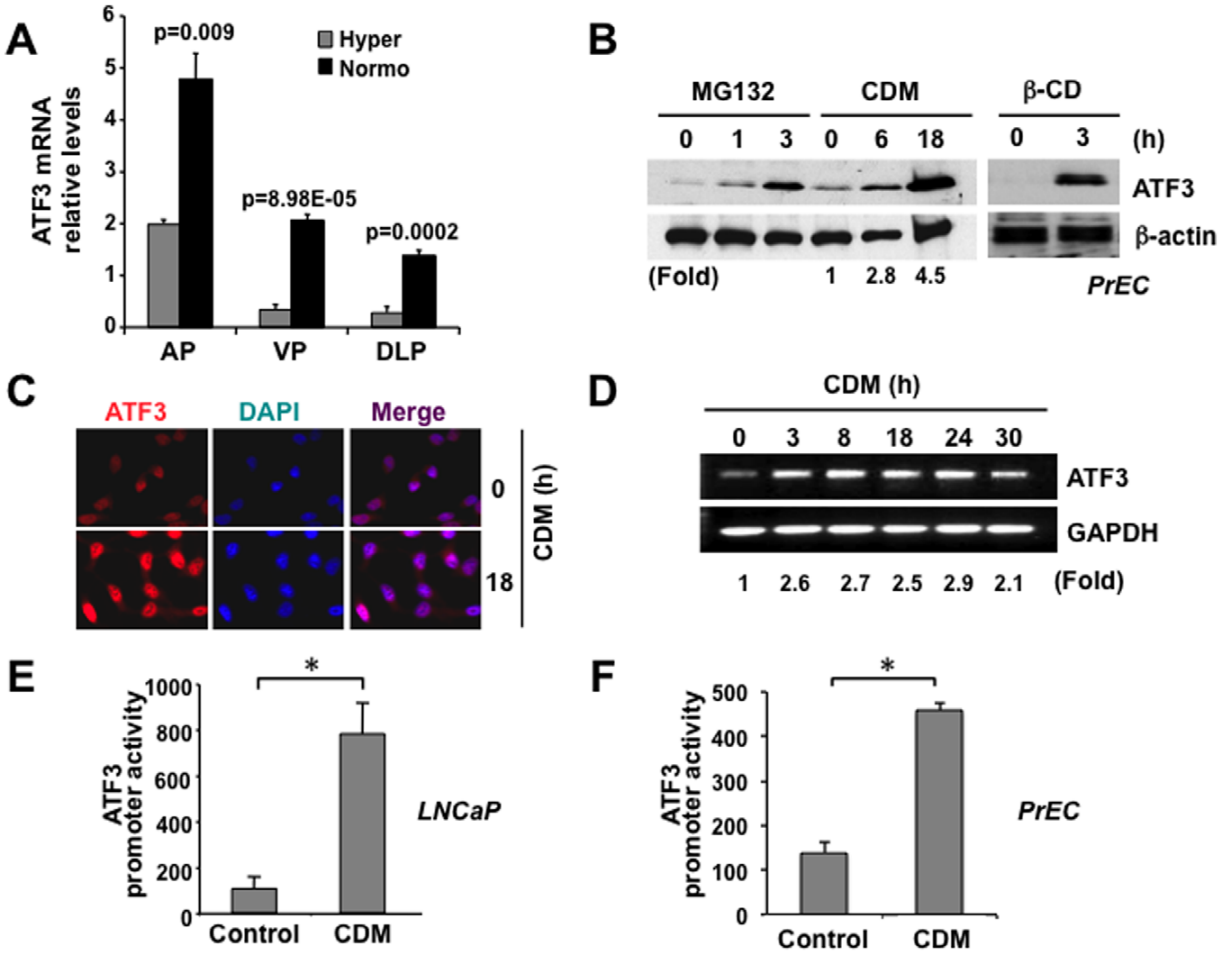
ATF3 expression coincides with reduced cholesterol. (**A**) **RT-PCR analysis** ***in vivo*** **.** ATF3 levels are reduced in all prostatic lobes from Hyper mice, compared to those from the Normo group (AP = anterior prostate; VP = ventral prostate; DLP = dorsal prostate). (**B**) **Immunoblot analysis.** Immunoblot of PrEC lysates showed induction of ATF3 protein by CDM (left panel) and by β-cyclodextrin (right panel). MG132, a proteasome inhibitor, also increased ATF3 expression. (**C**) **Immunofluorescence analysis.** Induction of ATF3 protein by CDM in LNCaP cells as shown by IF. LNCaP cells were treated with CDM for 18 h, stained with anti-ATF3 antibody and nuclei were counterstained with DAPI (left panel: ATF3; middle panel: DAPI; right panel: overlay). (**D**) **RT-PCR analysis.** ATF3 mRNA levels in LNCaP cells treated with CDM were normalized to levels of GAPDH. RT-PCR analysis shows induction of ATF3 mRNA levels by CDM. (**E–F**) **Promoter** **reporter analysis.** A full-length ATF3 promoter was cloned into a luciferase reporter vector and transfected into LNCaP (D) or PrEC (E). Cells were then incubated in Control and CDM medium. ATF3 promoter activity was plotted as arbitrary units (± SD) after normalization with total protein concentration.

SCID mice fed the hypercholesterolemic (Hyper) diet demonstrated ∼3 fold increased proliferation in prostate tissues, compared to the Normo condition, based on Ki67 staining index (Fig. 1A). As expected, Hyper mice showed ∼2.5 fold increase in serum cholesterol (Fig. 1B). Cholesterol level in the membrane fraction of mouse prostate tissues was also increased >1.5 fold in the high cholesterol cohort (Fig. 1C), indicating that elevated circulating cholesterol affects membrane lipid composition of prostate cells *in vivo.*

**Figure 5:**
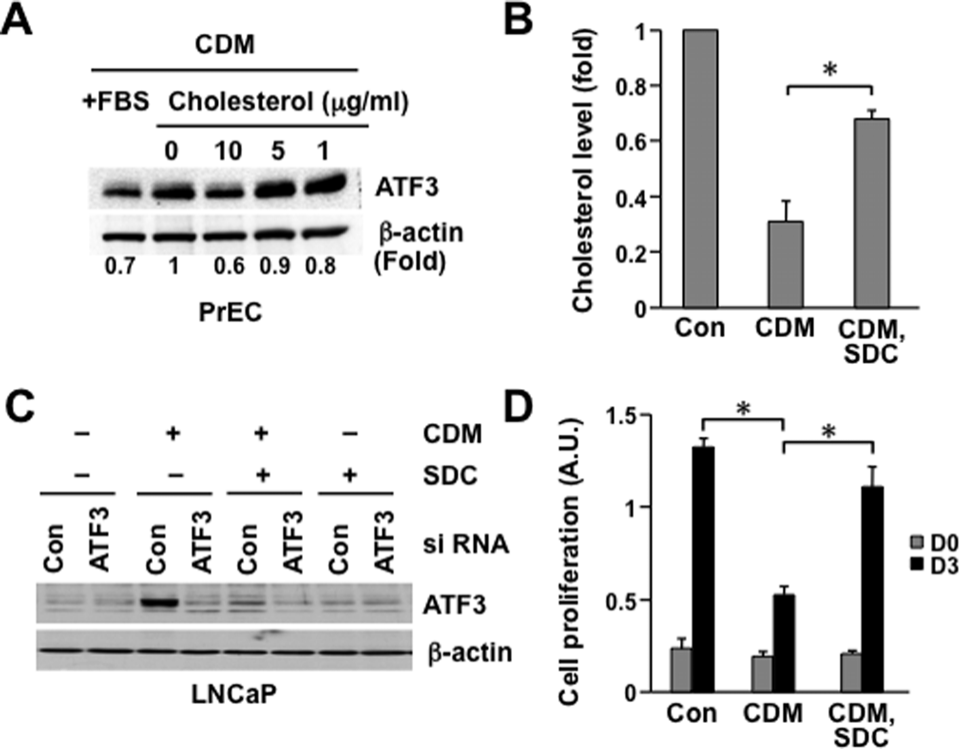
Cholesterol reduces ATF3 expression in prostate epithelial cells. (**A**) PrEC were incubated in CDM for 18 h and then water-soluble cholesterol (cholesterol loaded cyclodextrin) was added to the medium in a dose dependent manner (0, 1, 5, and 10 µg/ml) for 3h. After immunoblot analysis, band intensities were normalized to β-actin and fold changes are shown. (**B**) **Cholesterol-containing liposomes increase cholesterol level.** LNCaP cells were incubated in CDM for 18 h in the absence or presence of cholesterol containing liposomes (SDC (33% (mol) sphingomyelin+DOPC+cholesterol) [33]. Data are plotted as percent cholesterol level (± SD) (**C**) **Cholesterol containing liposomes reverse ATF3 protein induction by CDM.** LNCaP cells were transiently transfected with control siRNA (siCon) or siATF3. 48 h after transfection, cells were incubated in CDM for 18 h in the absence or presence of SDC. ATF3 protein levels were measured by immunoblot analysis. (**D**) **Enhanced proliferation by cholesterol containing liposomes.** LNCaP cells were incubated in CDM in the absence or presence of SDC [33]. After 3 d, cell proliferation was measured. Data are plotted as cell proliferation (A.U., absorption units) vs. time (days) ± SD (n = 3). Con, serum containing growth medium; CDM, cholesterol depletion medium; SDC, cholesterol containing liposome preparation. *p<0.05 (Student’s t-test).

To characterize molecular responses to the elevation in circulating cholesterol, gene expression profiling of SCID mouse prostate tissue was performed using Affymetrix mouse 430A.2 microarrays. We identified 1815 differentially expressed genes (DEGs) with a false discovery rate (FDR)<0.05. 877 DEGs were upregulated and 938 were downregulated in the ventral prostate (VP) lobes from the Hyper vs. Normo groups. GOBP and KEGG pathway enrichment analysis (Table S1) demonstrated that the upregulated genes were mainly involved in cellular processes related to cell proliferation, inflammation, and chemotaxis (p<0.05), while the downregulated genes were related to biosynthesis and cell adhesion, as well as protein folding, transport, localization, and degradation. Genes involved in inflammation/chemotaxis and cell proliferation/apoptosis suppression were largely upregulated (Table S1), consistent with the increased proliferation observed by direct examination of tissue (Fig. 1A).

**Figure 6:**
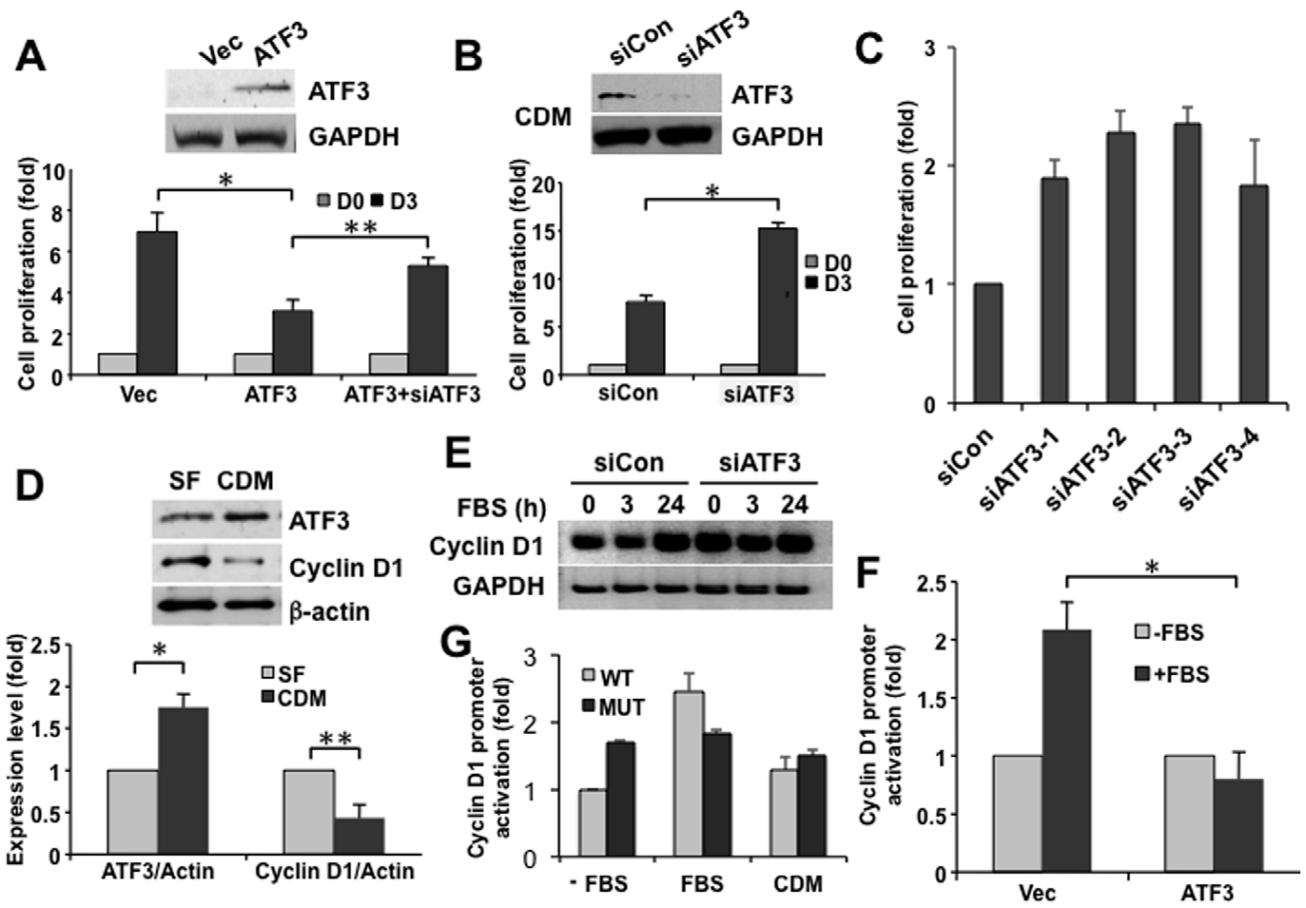
ATF3 is a negative regulator of cyclin D1 and cell proliferation. (**A**) **Enforced AFT3 expression reduces cell proliferation.** LNCaP cells were transiently transfected with an ATF3 expression construct, a vector alone control, or with the AFT3 expression construct + ATF3 siRNAs. Proliferation rate was measured at the indicated times. ATF3 expression levels were verified by immunoblot (upper panels). Data are plotted as cell proliferation (fold) vs. condition (Vec, ATF3, ATF3+siATF3) ± SD (n = 3). (**B–C**) **Knockdown of ATF3 increases cell proliferation.** LNCaP cells were transiently transfected with siATF3 (or siCon) and the number of cells at day 0 (grey bars) & day 3 (black bars) were measured. Four independent ATF3 siRNAs (siATF3-1, -2, -3, and -4) were transiently transfected, and cell numbers were determined at day 3. Data are plotted as cell proliferation (fold) vs. condition ± SD (n = 3). (**D**) **Effect of cholesterol depletion on ATF3 and cyclin D1 expression.** LNCaP cell were treated in serum free media (SF; grey bars) or with CDM (black bars) for 16 h and the level of ATF3 and cyclin D1 were determined. Data were normalized to β-actin from the same blots. Immunoblot data are representative of the immunoblot result used in densitometry. Data are plotted as expression level (fold) vs. condition ± SD (n = 3). (**E**) **ATF3 regulates cholesterol depletion-induced cyclin D1 expression (immunoblot analysis).** LNCaP cells were transiently transfected with siATF3 (or siCon). After serum starvation for 16 h, cells were stimulated with 10% serum for the indicated times. Immunoblot analysis was performed to determine cyclin D1 expression in ATF3 deficient cells. (**F**) **ATF3 regulates cyclin D1 expression (promoter reporter analysis).** LNCaP cells were transfected with promoter construct of cyclin D1 containing a luciferase reporter and followed by additional incubation with ± serum for 6 h. Data are plotted as promoter activation (fold) vs. condition ± SD (n = 3). (**G**) **Promoter activation of cyclin D1 upon cholesterol alteration requires ATF3 binding on promoter region (promoter reporter analysis).** LNCaP cells were transfected with a luciferase construct of a wild type (WT) or an ATF3 binding site mutated cyclin D1 (MUT) promoter. Promoter activity was measured 6 h after treatment with various conditions (±FBS or CDM). Data are plotted as promoter activation (fold) vs. condition ± SD (n = 4). All experiments were performed a minimum of 3 times. *p<0.05, **p<0.01 (Student’s t-test).

As an alternative approach to testing the effect of altered cholesterol availability on prostate cells, cholesterol levels were reduced in cultured human prostate cells using a published procedure employing acute exposure to cholesterol-depleted medium (hereafter referred to as CDM) [25]. In addition to using LDL-deficient serum, this medium includes an HMG-CoA reductase inhibitor to reduce endogenous cholesterol synthesis, along with a titrated amount of mevalonic acid to prevent depletion of non-sterol end products, e.g. isoprenoids. Treatment with CDM suppressed proliferation of both LNCaP prostate cancer cells (Fig. 2A) and normal human prostate epithelial cells (PrEC) (Fig. 2B), consistent with the essential role of cholesterol in cell proliferation. Incubating LNCaP cells in CDM resulted in a ≈60% reduction in intracellular cholesterol (Fig. 2C), but no cytotoxicity and no evidence of apoptosis above baseline was detected (Fig. 2D).

**Figure 7:**
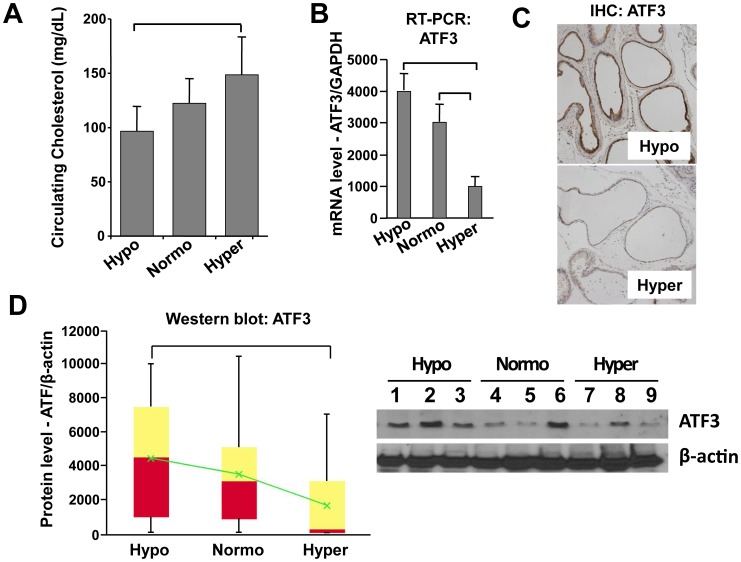
ATF3 expression level is associated with cholesterol level *in vivo*. Male C57BL/6 mice were fed Hypo, Normo, or Hyper diets for 4 months. (**A**) **Circulating cholesterol levels.** Serum cholesterol levels are plotted as cholesterol (mg/dL) vs. diet group ± SD (n = 18/group). (**B**) **RT-PCR analyses of ATF3 expression.** The expression levels of ATF3 mRNA and protein were compared in ventral prostate (VP) from male C57BL/6 mice in Hypo, Normo or Hyper conditions by quantitative densitometry. Data are plotted as mRNA level (arbitrary unit) vs. condition ± SD (n = 3). GAPDH expression was used to normalize gene expression. (**C**) **Immunohistochemical analysis of ATF3 expression.** Sections of VP tissues from mice in Hypo, Normo or Hyper groups, stained with anti-ATF3 antibody. Representative images of Hypo and Hyper are shown. (**D**) **Immunoblot analyses of ATF3**. Immunoblot data are presented as box and whisker plots of ATF3 expression levels (arbitrary units) vs. group. Bottom of red = median of lower half of the data. Top of yellow = median of upper half of the data. Intersection of red and yellow = median. Green = average. Vertical bars extend to maxima and minima (n =  18/group). *p<0.05 (two way ANOVA and Student’s t-test). Representative western blot data are shown (right).

Gene expression profiling of LNCaP cells exposed to CDM conditions for 0, 3 or 16 h was performed using Human Genome U133A 2.0 microarrays. We identified 217 and 2477 DEGs in the 3 and 16 h conditions, respectively (See Materials and Methods; Fig. S2A and S2B). Analysis of gene expression patterns revealed 8 distinct groups (Fig. S2B), with the largest groups of DEGs (groups 4 and 5) not differentially expressed until 16 h after initiation of the experiment, while the second largest groups (groups 1 and 8) were differentially expressed from 3 h and continued to 16 h. Functional enrichment analysis of DEGs (Table S2) shows that the up-regulated genes induced by CDM treatment were mainly involved in cellular processes related to the folding, transport, localization, and degradation of proteins (p<0.05), while the down-regulated genes were involved in processes related to cell cycle progression, cell proliferation, inflammation, and immune response, consistent with the finding that CDM medium suppressed rates of cell growth (Fig. 2A). The results in Tables S1 and S2 indicate that similar cellular processes were affected by hyper- and hypocholesterolemic (Hypo) conditions, but in opposite directions.

**Figure 8:**
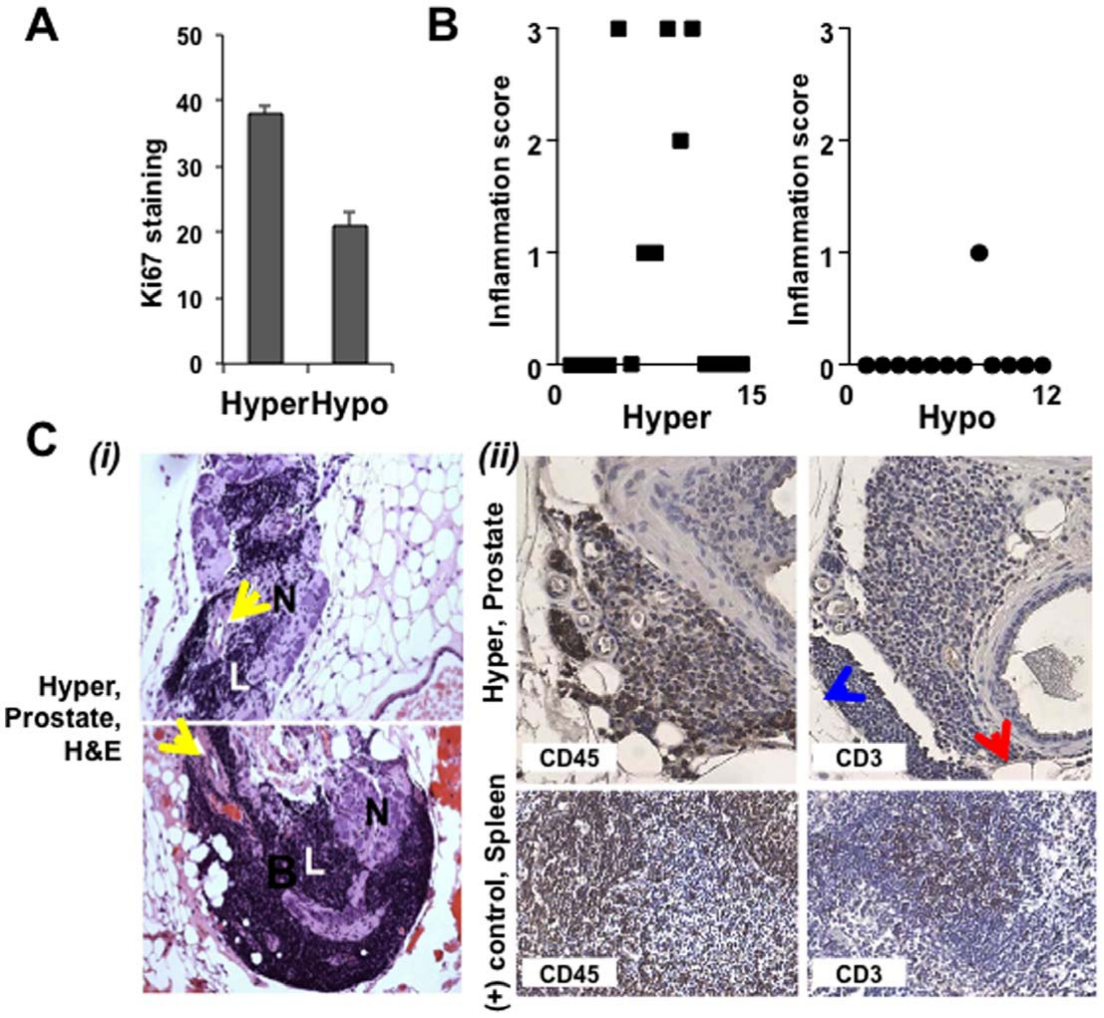
Hypocholesterolemia suppresses prostatic inflammation. (**A**) **Proliferative index.** Proliferating cells were counted by Ki-67 staining as described in Materials and Methods. Data are plotted as Ki-67 positive cells vs. condition. (**B**) **Inflammation score.** Infiltrating cells were scored as described in Materials and Methods. Data are plotted as inflammation score vs. condition. (**C-** ***i***) **VP lobes from male C57BL/6 mice in the Hyper condition.** Lymphoid cell populations (L) infiltrating periprostatic adipose tissue adjacent to nerves (N) and blood vessels were observed in the Hyper condition (yellow arrows). Two representative fields are shown. (**C-** ***ii***) IHC staining with anti-CD45 (1∶150) and anti-CD3 (1∶200) show inflammatory infiltrates observed in the Hyper condition are a mixture of B and T cells. Spleen tissue from male C57BL/6 mice was used as a positive control for IHC and protocol optimization. Blue arrowhead, adipose tissue; Red arrowhead, a prostatic acinus.

### A Cholesterol-sensitive Network

The overlap in the gene expression response to hypercholesterolemia in the mouse prostate and to CDM in LNCaP cells prompted us to integrate both data sets. We identified 449 genes altered in the 2 conditions (Fig. S3). These 449 DEGs were categorized into 4 groups based on the expression patterns between Hyper mouse prostates and CDM-treated LNCaP cells. This analysis narrowed the genes of interest to 265 (Clusters 2 and 3, Fig. S3A), whose expression levels were significantly altered in opposing directions in the 2 experimental settings. Using this gene set, we then constructed a hypothetical cholesterol-sensitive network to obtain a deeper and broader understanding of the genes/proteins underlying the prostatic response to changes in cholesterol.

The network (Fig. 3A) includes several interesting modules (clusters of functionally related genes based on GOBPs), including modules related to carbohydrate metabolism, inflammation, cell cycle regulation, cell migration, chromosome remodeling, RNA splicing, vesicular transport and steroid biosynthesis. The node and node boundary colors in Fig. 3A represent gene expression changes induced by hypercholesterolemia *in vivo* and by CDM treatment *in vitro*, respectively (See Methods or Figure legends). To assess the validity of this network, we used RT-PCR to assay for differential expression of a total of 8 genes, which we considered representative of all functional categories, in the *in vivo* Hyper vs. Normo conditions or in the cull culture CDM vs. normal medium conditions. Two genes with a high degree of centrality (See Materials and Methods), ATF3 (activating transcription factor 3, a mammalian ATF/CREB family transcription factor) and a known ATF3 target, ID1 (DNA-binding protein inhibitor 1), were tested under both conditions (Figs. 3B and 3C). As predicted from the microarray data, hypercholesterolemia reduced the mRNA levels of ATF3 as well as ID1, and enhanced those of C3 (apolipoprotein C3), S100A (S100 calcium-binding protein A) and IFI44 (gamma-interferon-inducible protein 44) (Fig. 3B). Also consistent with the microarray findings, in the cell culture setting mRNA levels of ATF3, ID1, ID2, CDK2 (cyclin-dependent kinase 2), and VEGFC (Vascular endothelial growth factor C) were up- (ATF3, ID1 and ID2) or down-regulated (CDK2 and VEGFC) in response to CDM treatment (Fig. 3C). These findings support the validity of the network model shown in Figure 3A.

### ATF3: a Central Node in the Prostatic Response to Changing Cholesterol Levels

To identify one or more “key” regulators of the prostatic response to cholesterol, we performed the following analysis. We selected genes for regulatory proteins (e.g. transcription factors, signaling molecules) from the 265 consistently altered DEGs. For each regulator, we then tallied its degree of centrality, based on the number of known DEG interactors (See Materials and Methods). The goal was to identify the key regulators with the largest degree of centrality in the network. This analysis identified ATF3 as one such potential key regulator because it has known targets ICA1 (islet cell autoantigen 1), ID1, TCF12 (transcription factor 12), TBL1X (transducin (beta)-like 1X-linked) and RNF14 (E3 ubiquitin-protein ligase) in at least 4 distinct modules (vesicular transport, cell proliferation, chromosome remodeling and RNA splicing) in the cholesterol-sensitive network.

ATF3 has been reported to participate in both cell proliferation and immunity [26], [27], and due to alternative splicing functions, can act as both an activator and repressor of gene transcription [28]–[32]. As a further test of the network model, we focused specifically on ATF3 and its sensitivity to cholesterol. RT-PCR analysis using three prostatic lobes (anterior prostate, AP; ventral prostate, VP and dorsolateral prostate, DLP) demonstrated that ATF3 gene expression levels were significantly decreased in the Hyper, compared to the Normo condition (Fig 4A). Consistent with these downward changes seen in hypercholesterolemia, expression of the ATF3 protein was induced in a time-dependent manner in both PrEC and LNCaP cells in response to cholesterol lowering in CDM (Figs. 4B and 4C). ATF3 was also acutely induced in response to rapid depletion of membrane cholesterol with methyl-β-cyclodextrin (β-CD), a cholesterol-absorbing reagent (Fig. 4B, right panel). ATF3 mRNA level was increased approximately 2.5-fold after 3 h of CDM treatment and this increase was retained for at least 30 h (Fig. 4D). Assessment of the responsiveness of the ATF3 promoter to cholesterol by luciferase reporter assay showed that CDM evoked ATF3 transcriptional activation in LNCaP (Fig. 4E) as well as PrEC (Fig. 4F), consistent with the mRNA and protein data.

In order to test whether adding endogenous cholesterol reverses the induction of ATF3 expression upon cholesterol depletion, human PrEC were incubated in CDM with and without soluble cholesterol (Fig. 5A). The presence of 10 µg/ml water-soluble cholesterol inhibited CDM-induced ATF3 expression approximately 40% (Fig. 5A). A cholesterol containing liposome preparation (SDC) [33], containing 33 mol% each of sphingomyelin, 1,2-Dioleoyl-*sn*-glycero-3-phosphocholine (DOPC) and cholesterol, raised cholesterol level in LNCaP cells (Fig. 5B) and reversed ATF3 upregulation induced by CDM treatment (Fig. 5C). Significantly, this manipulation reversed the inhibition of cell proliferation induced by CDM (Fig. 5D).

These data show that ATF3 expression is regulated by cholesterol level both in normal immortalized prostate epithelial cells and in androgen-dependent prostate cancer cells. Moreover, the inverse correlation of ATF3 level and cell proliferation suggests that altered ATF3 may mediate the effect of cholesterol on regulating cell proliferation.

### ATF3 Regulates Prostate Cell Proliferation

We next performed functional studies in LNCaP cells to assess the biological role of ATF3. Enforced expression of ATF3 inhibited cell proliferation, an effect that was reversed by ATF3-targeted RNA interference (RNAi) (Fig. 6A). Conversely, knockdown of ATF3 using RNAi increased cell growth (Fig. 6B), suggesting that the endogenous protein is growth suppressive. ATF3 levels were low in growing cells without added stimuli, requiring CDM treatment for visualization (Fig. 6B, upper panel). To rule out the off-target effects of siRNAs, 4 independent ATF3 siRNAs were tested. Consistent with results using the ATF3 siRNA pool, cell proliferation was enhanced when ATF3 expression was depleted by the 4 individual ATF3 siRNAs (Fig. 6C).

Notably, the increased ATF3 level (>1.5 fold) in response to CDM coincided with reduced cyclin D1 expression (≈0.5 fold compared to time 0) (Fig. 6D). Because cyclin D1 plays an essential role in the cell cycle transition from early to mid-G1 phases, we asked whether there is a functional link between ATF3 and cyclin D1 in the context of cell proliferation. Fetal bovine serum (10%) raised cyclin D1 expression as expected in a time-dependent manner in serum-starved control cells. ATF3 knockdown by RNAi increased cyclin D1 levels even in the absence of serum stimulation (Fig. 6E). Cyclin D1 promoter-driven expression of luciferase was also enhanced in response to serum, and was almost completely suppressed by ATF3 overexpression (Fig. 6F). To determine whether cyclin D1 mediates proliferative effects of ATF3 in response to cholesterol alterations, we compared the response of the wild type cyclin D1 (WT) promoter, and an identical promoter with the ATF3-binding site inactivated by mutation (MUT), after incubation in two control conditions (medium ± FBS) or CDM. Cyclin D1 promoter activation in response to cholesterol alteration was diminished when the ATF3 binding site was mutated (Fig. 6G). These results imply that ATF3 downregulation under conditions of hypercholesterolemia may activate cyclin D1 expression and lead to enhanced cell proliferation.

### Influence of Cholesterol on ATF3 Expression and Inflammatory Infiltrates

In order to further investigate whether circulating cholesterol levels alter ATF3 expression *in vivo* in immune-intact animals, 7–8 wk old male C57BL/6 mice (n = 18/group) were maintained under Hypo, Normo or Hyper conditions for 4 months. There were no significant differences in weight, insulin level, circulating testosterone/DHT level, and no detectable liver dysfunction, in the 2 groups. The Hypo diet resulted in circulating cholesterol levels about 60% lower than the Hyper diet (p<0.05), and the 3 diet regimens resulted in 3 distinct average cholesterol levels (Fig. 7A). To assess the effect of circulating cholesterol level on ATF3 expression, we measured ATF3 mRNA and protein levels in C57BL/6 mouse prostate tissue under Hypo, Normo or Hyper conditions. Consistent with the findings presented above, ATF3 mRNA (Fig. 7B) and protein (Fig. 7C and 7D) were markedly reduced, seemingly in a step-wise manner, with increasing cholesterol.

Prostate tissue from Hypo mice showed evidence of substantially decreased Ki67 staining (Fig. 8A) and lymphomononuclear cell accumulation (Fig. 8B), in comparison to the Normo (not shown) and Hyper groups, indicating that cholesterol reduction may reduce prostatic inflammation. In the Normo and Hyper groups, inflammatory cell clusters (L) were found closely associated with nerve tracts (N) and blood vessels (yellow arrows) in Hyper animals (Fig. 8C *(i)*). The presence of lymphomononuclear infiltrates was negligible in the Hypo condition (data not shown). IHC analysis using marker proteins of immune cells (CD45, a general marker for lymphocytes; CD3, T cell marker) revealed that the monocytoid cells in the infiltrates are a mixture of B and T cells, with a predominance of B lymphocytes (Fig. 8C *(ii)*).

## Discussion

In this study, we sought to gain insight into the mechanism by which the normal prostate responds to changes in cholesterol level in the microenvironment. We took a systems approach of identifying a cholesterol-responsive gene set and constructing a signaling network. We then tested this network using a series of independent approaches. We used an isocaloric diet method that allows isolation of cholesterol level as a variable independent of energy effects, animal weight variation, level of visceral fat or changes in circulating androgen [7]. Direct examination of prostate tissue from mice kept under chronic hypercholesterolemia demonstrated increased cell proliferation and under hypocholesterolemic conditions showed suppressed inflammatory cell infiltration. These findings suggest that circulating cholesterol may trigger pathophysiological changes in the prostate, and that lowering cholesterol may inhibit such changes. *In vitro* cholesterol depletion experiments showed that cell proliferation is inhibited by insufficient cellular cholesterol. By integrating unbiased gene expression data from the *in vivo* and *in vitro* experiments, we identified a network of cholesterol-regulated genes in the prostate. Further analysis indicated that the transcription factor ATF3 is likely to be an important node in this network. To our knowledge, this is the first systematic study of the signaling mechanisms in the prostate affected by changes in cholesterol levels.

ATF3 is a 21kDa leucine zipper (bZIP) transcription factor that belongs to the ATF/CREB protein family. ATF3 mRNA levels are low or undetectable in most tissues under most conditions, but are induced by a variety of environmental cues, including cytokines, xenotoxic agents and physiological stresses [26]. ATF3 has been identified as a suppressor of innate immunity, viral immunity, and allergic inflammation [26], [34]–[38]. ATF3 suppresses LPS-induced expression of interleukin (IL)-6 and IL-12 in macrophages by working in conjunction and in opposition to Rel (an NFκB subunit), an activator of IL-6 and -12 genes [37]. ATF3 is also activated in the lungs of mice after allergen challenge, where it contributes to negative regulation of the expression of IL-4, IL-5, IL-13 and other CCL (β-chemokine ligand) chemokine genes that mediate immune cell infiltration to pulmonary tissue. Consequently, ATF3-deficient mice exhibit increased airway hypersensitivity including enhanced eosinophilia [36]. Together, those studies suggest that ATF3 regulates inflammatory responses.

As a transcriptional repressor of cyclin D1, IRS (insulin receptor substrate) 2, and ID1 expression in chondrocytes, mouse embryonic fibroblasts, mouse pancreatic beta cells, and HaCaT human skin keratinocyte cells, ATF3 functions as an anti-proliferative factor [39]–[43]. In cancer cells, ATF3 has also been identified as an inhibitor of Ras-stimulated tumorigenesis [43]. Those studies suggest that ATF3 induction correlates with cell cycle arrest, growth inhibition, and/or apoptosis. In contrast, ATF3 overexpression enhanced transcription of FN (fibronectin)-1, TWIST (twist transcription factor)-1 and Slug in MCF10CA1 breast cancer cells [44], and led to increased proliferation of DU145 prostate cancer cells [45]. Moreover, targeting ATF3 for knockdown inhibits cell adhesion and invasion capability in HT29 human colon cancer cells [46]. ATF3 increased apoptosis in untransformed MCF10A mammary epithelial cells, while it inhibited apoptosis in MCF10A breast tumor cells [44]. These discrepancies in function suggest that the biological role of ATF3 is highly dependent on physiologic conditions. The role of ATF3 in malignancy cannot be generalized as either being oncogenic or tumor suppressing; it exhibits properties of both functional classes. This duality of function has been explained as a consequence of: (a) cellular context; (b) post-translational modifications; (c) interacting proteins; (d) sub-cellular compartmentalization; and/or (e) differential complex formation of ATF3 homo- or heterodimers with other bZip proteins (e.g. c-Jun, Jun B and Jun D).

Hypocholesterolemic mice exhibited substantially reduced levels of inflammatory infiltrates in the prostate and relatively high expression of ATF3. Similarly, ATF3 mRNA level was rapidly increased by depriving cells of cholesterol *in vitro*, an effect that was sustained for at least 30 h (Fig. 4C). These data could be evidence for suppression by high cholesterol of a feedback regulatory mechanism that inhibits prostatic inflammation, although the present data do not provide a mechanistic explanation for how altered ATF3 expression might affect immune cell infiltration. One possibility is that chronic hypercholesterolemia may activate prostatic cells or/and adipocytes, through regulation of ATF3 levels, to secrete chemo-attractants (e.g. cytokines and chemotactic adipokines) that recruit immune cells.

Notably, dense inflammatory infiltrates were found adjacent to nerves, suggesting the possibility that prolonged high cholesterol might lead to chronic pain. Recruitment of inflammatory cells into nerve fibers has been identified as a likely source of visceral pain in chronic pancreatitis, and painful ejaculation symptoms occur in 5–31% of men with LUTS linked to BPH [47], [48]. In light of our present data, these observations, suggest the possibility that prolonged high cholesterol may promote symptoms associated with benign prostatic disease.

Our cholesterol sensitive network model revealed alterations in an inflammatory signature in response to variation of cholesterol level (Fig. 3A): Expression of S100A, C3 and IFI44 was increased in the Hyper group, and decreased in the Hypo condition (Fig. 3). Pathway analysis suggested that the immune/inflammation-related genes were significantly enriched in the Hyper condition (Table S1), suggesting an inflammatory response to high cholesterol. Accumulating evidence has linked pathologic or premalignant changes in the prostate with chronic inflammation. In particular, most BPH tissues show evidence of a chronic inflammatory reaction: (a) only 23% of prostate biopsies from 284 BPH patients were free of infiltrating inflammatory cells [49], (b) the presence of inflammatory infiltrates in BPH tissues was associated with increased rates of disease progression and elevated risk of acute urinary retention (Medical Therapy of Prostatic Symptoms (MTOPS) study), (c) human BPH stromal cells isolated from surgical specimens express all of the toll-like receptors (TLRs), which are central mediators of the innate immune system [50]; and (d) a recent report of a BPH signaling network suggested activation of inflammation through the TGFβ-Smad2/3 signaling pathway [51]. Chronic inflammatory infiltrates in BPH nodules are mainly composed of T cells and macrophages [52]–[54], which are recruited to the prostate by chemoattractants, including IL-6, IL-8 and IL-15. In particular, IL-8 is highly expressed in BPH specimens [55] and stimulates prostatic stromal and epithelial growth during BPH progression [56].

In summary, we have identified elements of a cholesterol-sensitive network in the normal prostate, thereby demonstrating that the prostate senses and can mount a response to chronic changes in circulating cholesterol. Because cholesterol-lowering drugs of several types are in widespread use, further study of the effects of cholesterol on the prostate and on lower urinary tract function may uncover novel therapeutic strategies.

## Supporting Information

Figure S1 **Inflammation was observed in H&E staining from C57BL6 mice, but not in SCID mice, in normal diet condition.**
(TIF)

Figure S2 **Nucleotide microarray constructed gene clusters based on expression pattern.** LNCaP prostate cells were treated with CDM for 0, 3 or 16h, and RNAs extracted were used for RNA profiling. Gene expression levels of LNCaP cells at 3 or 16 h of treatment with CDM in comparison with untreated cells are displayed. Green: downregulated genes; red: upregulated genes in response to exposure to CDM. **(A) Heat map showing up- or down-regulated genes by cholesterol depletion. (B) 8 clusters of transcripts were identified based on expression pattern.** The largest cluster containing proliferation-related genes showed a marked downregulation by CDM.
(TIF)

Figure S3 **Integration of two DNA microarray datasets.** Two separately acquired microarray data from mouse prostate (*in vivo*) and LNCaP cells (*in vitro)* were integrated to extract the emerging cholesterol-sensing gene network in prostate cells in response to cholesterol manipulation. **(A)** 449 genes were identified in the Hyper and CDM conditions and categorized into four groups: (1) both up, (2) up in one or the other, and (3) both down. We focused the 265 genes that were significantly altered in opposite directions by Hyper and CDM. **(B) Heat map showing the commonly found genes in Hyper and Hypo (CLM) condition.** Green, downregulated genes; Red, upregulated genes compared to control.
(TIF)

Table S1 **Enriched GOBP or KEGG terms by DEGs affected by Hyper diet in the SCID VP lobe.**
(TIF)

Table S2 **Enriched GOBP or KEGG terms by DEGs affected by CDM in LNCaP cells.**
(TIF)

## References

[1] Moyad MA, Lowe FC (2008). Educating patients about lifestyle modifications for prostate health.. Am J Med.

[2] de Ferranti S, Mozaffarian D (2008). The perfect storm: obesity, adipocyte dysfunction, and metabolic consequences.. Clin Chem.

[3] Marcovecchio M, Mohn A, Chiarelli F (2005). Type 2 diabetes mellitus in children and adolescents.. J Endocrinol Invest.

[4] Qi L, Saberi M, Zmuda E, Wang Y, Altarejos J (2009). Adipocyte CREB promotes insulin resistance in obesity.. Cell Metab.

[5] Freeman MR, Solomon KR (2004). Cholesterol and prostate cancer.. J Cell Biochem.

[6] Zhuang L, Kim J, Adam RM, Solomon KR, Freeman MR (2005). Cholesterol targeting alters lipid raft composition and cell survival in prostate cancer cells and xenografts.. J Clin Invest.

[7] Solomon KR, Pelton K, Boucher K, Joo J, Tully C (2009). Ezetimibe is an inhibitor of tumor angiogenesis.. Am J Pathol.

[8] Mostaghel EA, Solomon KR, Pelton K, Freeman MR, Montgomery RB (2012). Impact of circulating cholesterol levels on growth and intratumoral androgen concentration of prostate tumors.. PLoS One.

[9] Graaf MR, Beiderbeck AB, Egberts AC, Richel DJ, Guchelaar HJ (2004). The risk of cancer in users of statins.. J Clin Oncol.

[10] Murtola TJ, Tammela TL, Lahtela J, Auvinen A (2007). Cholesterol-lowering drugs and prostate cancer risk: a population-based case-control study.. Cancer Epidemiol Biomarkers Prev.

[11] Flick ED, Habel LA, Chan KA, Van Den Eeden SK, Quinn VP (2007). Statin use and risk of prostate cancer in the California Men's Health Study cohort.. Cancer Epidemiol Biomarkers Prev.

[12] Platz EA, Clinton SK, Giovannucci E (2008). Association between plasma cholesterol and prostate cancer in the PSA era.. Int J Cancer.

[13] Parsons JK, Bergstrom J, Barrett-Connor E (2008). Lipids, lipoproteins and the risk of benign prostatic hyperplasia in community-dwelling men.. BJU Int.

[14] Pelton K, Di Vizio D, Insabato L, Schaffner CP, Freeman MR (2010). Ezetimibe reduces enlarged prostate in an animal model of benign prostatic hyperplasia.. J Urol.

[15] Adam RM, Danciu T, McLellan DL, Borer JG, Lin J (2003). A nuclear form of the heparin-binding epidermal growth factor-like growth factor precursor is a feature of aggressive transitional cell carcinoma.. Cancer Res.

[16] Kim J, Adam RM, Freeman MR (2005). Trafficking of nuclear heparin-binding epidermal growth factor-like growth factor into an epidermal growth factor receptor-dependent autocrine loop in response to oxidative stress.. Cancer Res.

[17] Kim J, Jahng WJ, Di Vizio D, Lee JS, Jhaveri R (2007). The phosphoinositide kinase PIKfyve mediates epidermal growth factor receptor trafficking to the nucleus.. Cancer Res.

[18] Berger R, Febbo PG, Majumder PK, Zhao JJ, Mukherjee S (2004). Androgen-induced differentiation and tumorigenicity of human prostate epithelial cells.. Cancer Res.

[19] Adam RM, Mukhopadhyay NK, Kim J, Di Vizio D, Cinar B (2007). Cholesterol sensitivity of endogenous and myristoylated Akt.. Cancer Res.

[20] Storey JD, Tibshirani R (2003). Statistical significance for genomewide studies.. Proc Natl Acad Sci U S A.

[21] Hwang D, Rust AG, Ramsey S, Smith JJ, Leslie DM (2005). A data integration methodology for systems biology.. Proc Natl Acad Sci U S A.

[22] Lempicki RA, Polis MA, Yang J, McLaughlin M, Koratich C (2006). Gene expression profiles in hepatitis C virus (HCV) and HIV coinfection: class prediction analyses before treatment predict the outcome of anti-HCV therapy among HIV-coinfected persons.. J Infect Dis.

[23] Junker BH, Koschutzki D, Schreiber F (2006). Exploration of biological network centralities with CentiBiN.. BMC Bioinformatics.

[24] Bergmann S, Ihmels J, Barkai N (2004). Similarities and differences in genome-wide expression data of six organisms.. PLoS Biol.

[25] Dong P, Flores J, Pelton K, Solomon KR (2010). Prohibitin is a cholesterol-sensitive regulator of cell cycle transit.. J Cell Biochem.

[26] Thompson MR, Xu D, Williams BR (2009). ATF3 transcription factor and its emerging roles in immunity and cancer.. J Mol Med.

[27] Hai T, Wolford CC, Chang YS (2010). ATF3, a hub of the cellular adaptive-response network, in the pathogenesis of diseases: is modulation of inflammation a unifying component?. Gene Expr.

[28] Hua B, Tamamori-Adachi M, Luo Y, Tamura K, Morioka M (2006). A splice variant of stress response gene ATF3 counteracts NF-kappaB-dependent anti-apoptosis through inhibiting recruitment of CREB-binding protein/p300 coactivator.. J Biol Chem.

[29] Pan Y, Chen H, Siu F, Kilberg MS (2003). Amino acid deprivation and endoplasmic reticulum stress induce expression of multiple activating transcription factor-3 mRNA species that, when overexpressed in HepG2 cells, modulate transcription by the human asparagine synthetase promoter.. J Biol Chem.

[30] Wang J, Cao Y, Steiner DF (2003). Regulation of proglucagon transcription by activated transcription factor (ATF) 3 and a novel isoform, ATF3b, through the cAMP-response element/ATF site of the proglucagon gene promoter.. J Biol Chem.

[31] Hashimoto Y, Zhang C, Kawauchi J, Imoto I, Adachi MT (2002). An alternatively spliced isoform of transcriptional repressor ATF3 and its induction by stress stimuli.. Nucleic Acids Res.

[32] Chen BP, Liang G, Whelan J, Hai T (1994). ATF3 and ATF3 delta Zip. Transcriptional repression versus activation by alternatively spliced isoforms.. J Biol Chem.

[33] Saslowsky DE, Lawrence JC, Henderson RM, Edwardson JM (2003). Syntaxin is efficiently excluded from sphingomyelin-enriched domains in supported lipid bilayers containing cholesterol.. J Membr Biol.

[34] Whitmore MM, Iparraguirre A, Kubelka L, Weninger W, Hai T (2007). Negative regulation of TLR-signaling pathways by activating transcription factor-3.. J Immunol.

[35] Rosenberger CM, Clark AE, Treuting PM, Johnson CD, Aderem A (2008). ATF3 regulates MCMV infection in mice by modulating IFN-gamma expression in natural killer cells.. Proc Natl Acad Sci U S A.

[36] Gilchrist M, Henderson WR, Clark AE, Simmons RM, Ye X (2008). Activating transcription factor 3 is a negative regulator of allergic pulmonary inflammation.. J Exp Med.

[37] Gilchrist M, Thorsson V, Li B, Rust AG, Korb M (2006). Systems biology approaches identify ATF3 as a negative regulator of Toll-like receptor 4.. Nature.

[38] Suganami T, Yuan X, Shimoda Y, Uchio-Yamada K, Nakagawa N (2009). Activating transcription factor 3 constitutes a negative feedback mechanism that attenuates saturated Fatty acid/toll-like receptor 4 signaling and macrophage activation in obese adipose tissue.. Circ Res.

[39] James CG, Woods A, Underhill TM, Beier F (2006). The transcription factor ATF3 is upregulated during chondrocyte differentiation and represses cyclin D1 and A gene transcription.. BMC Mol Biol.

[40] Li D, Yin X, Zmuda EJ, Wolford CC, Dong X (2008). The repression of IRS2 gene by ATF3, a stress-inducible gene, contributes to pancreatic beta-cell apoptosis.. Diabetes.

[41] Kashiwakura Y, Ochiai K, Watanabe M, Abarzua F, Sakaguchi M (2008). Down-regulation of inhibition of differentiation-1 via activation of activating transcription factor 3 and Smad regulates REIC/Dickkopf-3-induced apoptosis.. Cancer Res.

[42] Kang Y, Chen CR, Massague J (2003). A self-enabling TGFbeta response coupled to stress signaling: Smad engages stress response factor ATF3 for Id1 repression in epithelial cells.. Mol Cell.

[43] Lu D, Wolfgang CD, Hai T (2006). Activating transcription factor 3, a stress-inducible gene, suppresses Ras-stimulated tumorigenesis.. J Biol Chem.

[44] Yin X, Dewille JW, Hai T (2008). A potential dichotomous role of ATF3, an adaptive-response gene, in cancer development.. Oncogene.

[45] Pelzer AE, Bektic J, Haag P, Berger AP, Pycha A (2006). The expression of transcription factor activating transcription factor 3 in the human prostate and its regulation by androgen in prostate cancer.. J Urol.

[46] Ishiguro T, Nagawa H (2001). ATF3 gene regulates cell form and migration potential of HT29 colon cancer cells.. Oncol Res.

[47] Nickel JC, Elhilali M, Vallancien G (2005). Benign prostatic hyperplasia (BPH) and prostatitis: prevalence of painful ejaculation in men with clinical BPH.. BJU Int.

[48] Rosen R, Altwein J, Boyle P, Kirby RS, Lukacs B (2003). Lower urinary tract symptoms and male sexual dysfunction: the multinational survey of the aging male (MSAM-7).. Eur Urol.

[49] Morote J, Lopez M, Encabo G, de Torres IM (2000). Effect of inflammation and benign prostatic enlargement on total and percent free serum prostatic specific antigen.. Eur Urol.

[50] Penna G, Fibbi B, Amuchastegui S, Cossetti C, Aquilano F (2009). Human benign prostatic hyperplasia stromal cells as inducers and targets of chronic immuno-mediated inflammation.. J Immunol.

[51] Kim J, Yanagihara Y, Kikugawa T, Ji M, Tanji N (2009). A signaling network in phenylephrine-induced benign prostatic hyperplasia.. Endocrinology.

[52] Kramer G, Marberger M (2006). Could inflammation be a key component in the progression of benign prostatic hyperplasia?. Curr Opin Urol.

[53] Nickel JC (2008). Inflammation and benign prostatic hyperplasia.. Urol Clin North Am 35: 109–115; vii.

[54] Fibbi B, Penna G, Morelli A, Adorini L, Maggi M (2010). Chronic inflammation in the pathogenesis of benign prostatic hyperplasia.. Int J Androl.

[55] Konig JE, Senge T, Allhoff EP, Konig W (2004). Analysis of the inflammatory network in benign prostate hyperplasia and prostate cancer.. Prostate.

[56] Castro P, Xia C, Gomez L, Lamb DJ, Ittmann M (2004). Interleukin-8 expression is increased in senescent prostatic epithelial cells and promotes the development of benign prostatic hyperplasia.. Prostate.

