# Correction: The Response of the Prostate to Circulating Cholesterol: Activating Transcription Factor 3 (ATF3) as a Prominent Node in a Cholesterol-Sensing Network

**DOI:** 10.1371/annotation/b77e9022-f87c-4f77-9f82-9072351e3360

**Published:** 2012-08-16

**Authors:** Jayoung Kim, Dolores Di Vizio, Taek-Kyun Kim, Jonghwan Kim, Minjung Kim, Kristine Pelton, Steven K. Clinton, Tsonwin Hai, Daehee Hwang, Keith R. Solomon, Michael R. Freeman

There was an error in the citation. The correct citation is:

Kim J, Di Vizio D, Kim T-K, Kim J, Kim M, et al. (2012) The Response of the Prostate to Circulating Cholesterol: Activating Transcription Factor 3 (ATF3) as a Prominent Node in a Cholesterol-Sensing Network. PLoS ONE 7(7): e39448. doi:10.1371/journal.pone.0039448 

