# Supplementary figures and images for: The Response of the Prostate to Circulating Cholesterol: Activating Transcription Factor 3 (ATF3) as a Prominent Node in a Cholesterol-Sensing Network
Source: PLoS One. 2012 Jul 2;7(7):e39448. doi: 10.1371/journal.pone.0039448 (PMC3388073; doi:10.1371/journal.pone.0039448)

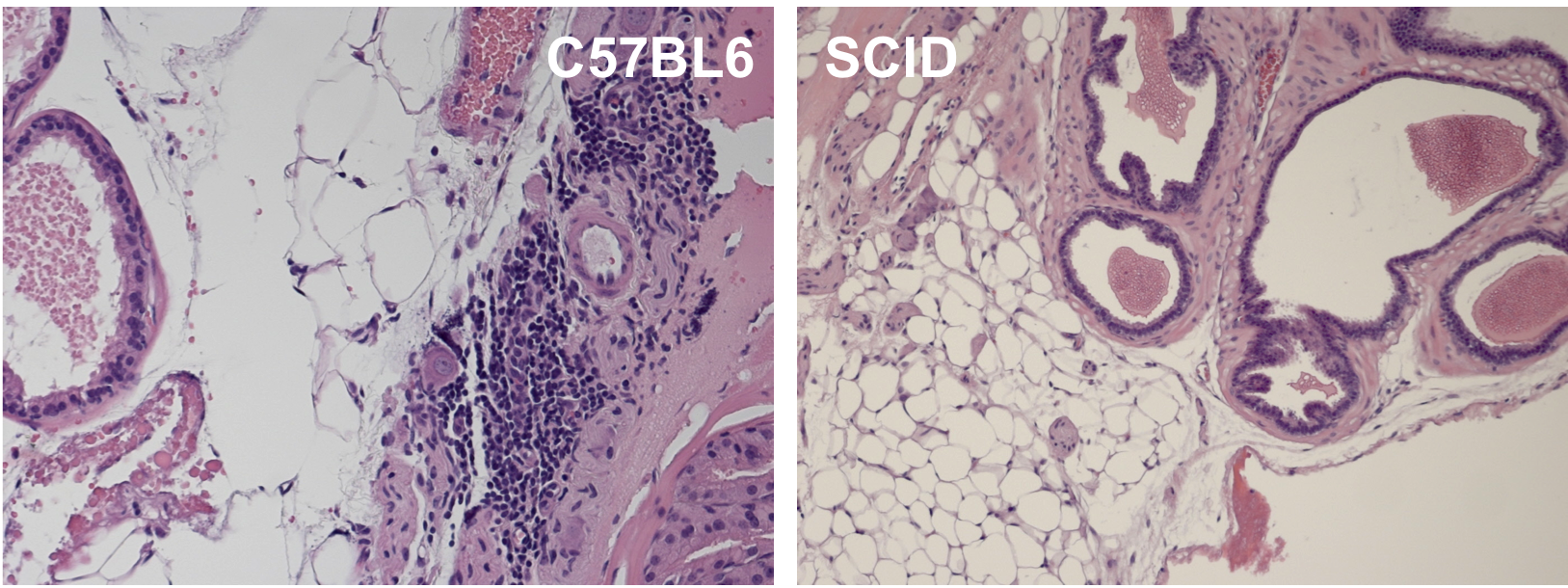

Supplement: Figure S1 — Inflammation was observed in H&E staining from C57BL6 mice, but not in SCID mice, in normal diet condition. (TIF) [file pone.0039448.s001.tif]

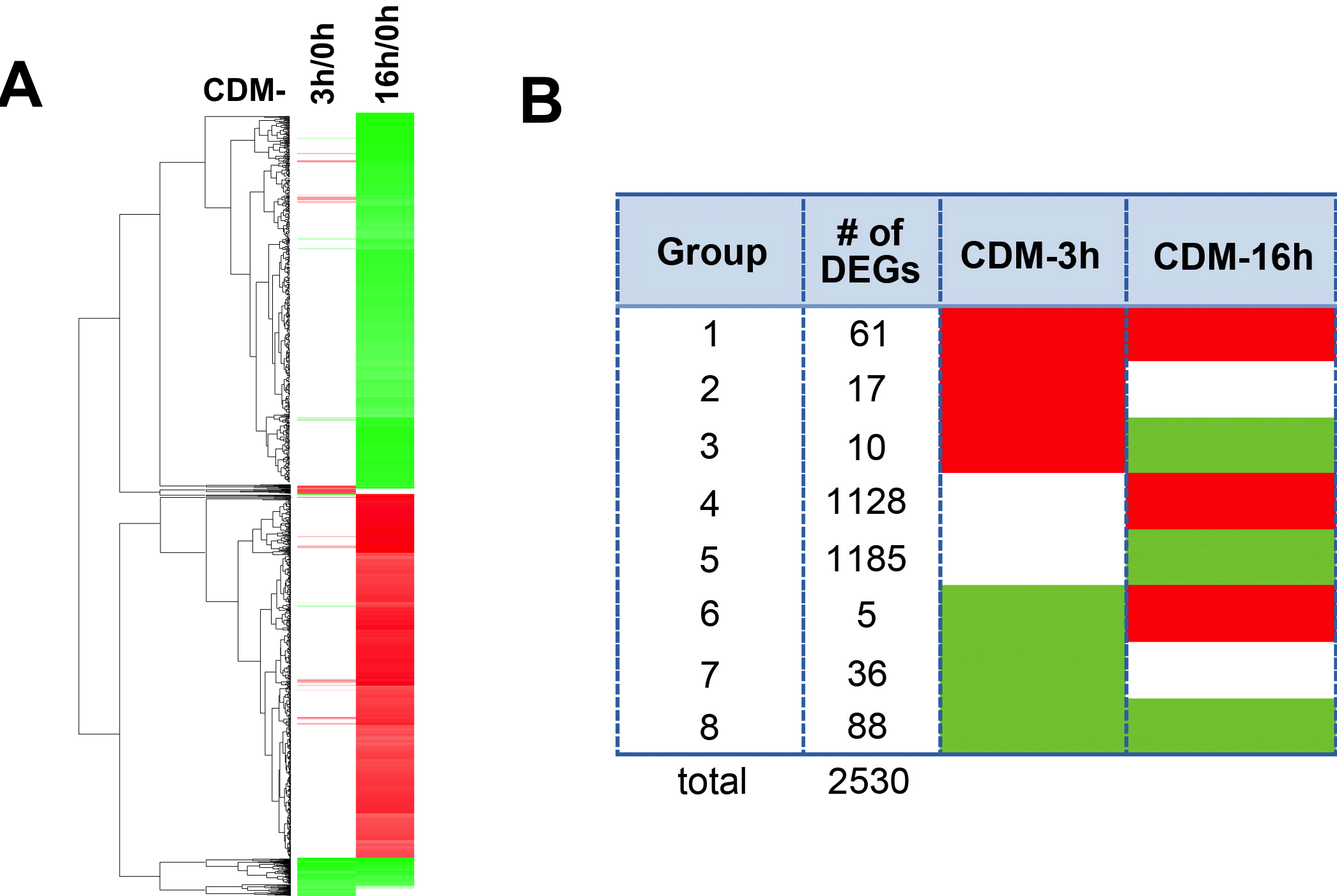

Supplement: Figure S2 — Nucleotide microarray constructed gene clusters based on expression pattern. LNCaP prostate cells were treated with CDM for 0, 3 or 16h, and RNAs extracted were used for RNA profiling. Gene expression levels of LNCaP cells at 3 or 16 h of treatment with CDM in comparison with untreated cells are displayed. Green: downregulated genes; red: upregulated genes in response to exposure to CDM. (A) Heat map showing up- or down-regulated genes by cholesterol depletion. (B) 8 clusters of transcripts were identified based on expression pattern. The largest cluster containing proliferation-related genes showed a marked downregulation by CDM. (TIF) [file pone.0039448.s002.tif]

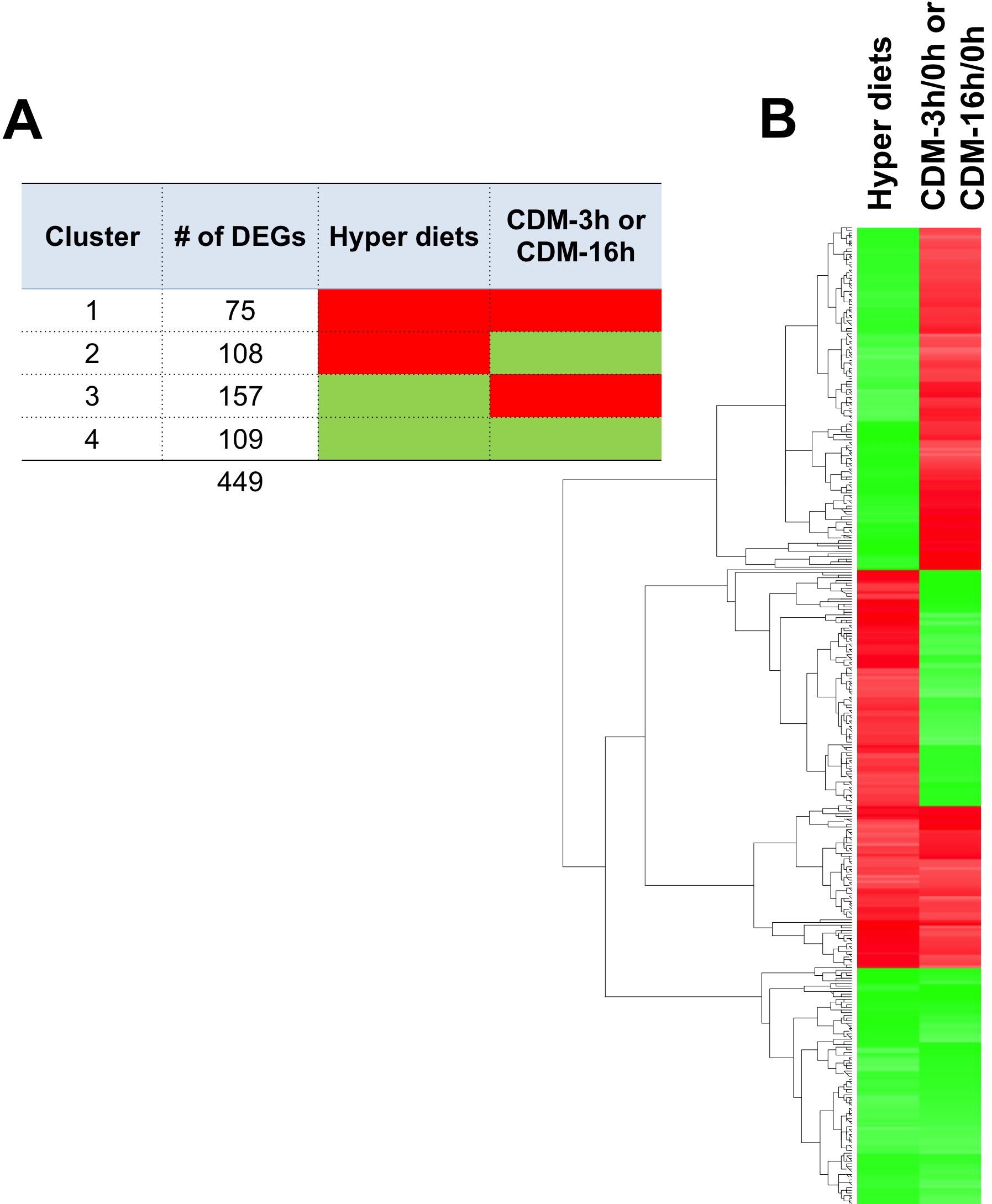

Supplement: Figure S3 — Integration of two DNA microarray datasets. Two separately acquired microarray data from mouse prostate (in vivo) and LNCaP cells (in vitro) were integrated to extract the emerging cholesterol-sensing gene network in prostate cells in response to cholesterol manipulation. (A) 449 genes were identified in the Hyper and CDM conditions and categorized into four groups: (1) both up, (2) up in one or the other, and (3) both down. We focused the 265 genes that were significantly altered in opposite directions by Hyper and CDM. (B) Heat map showing the commonly found genes in Hyper and Hypo (CLM) condition. Green, downregulated genes; Red, upregulated genes compared to control. (TIF) [file pone.0039448.s003.tif]

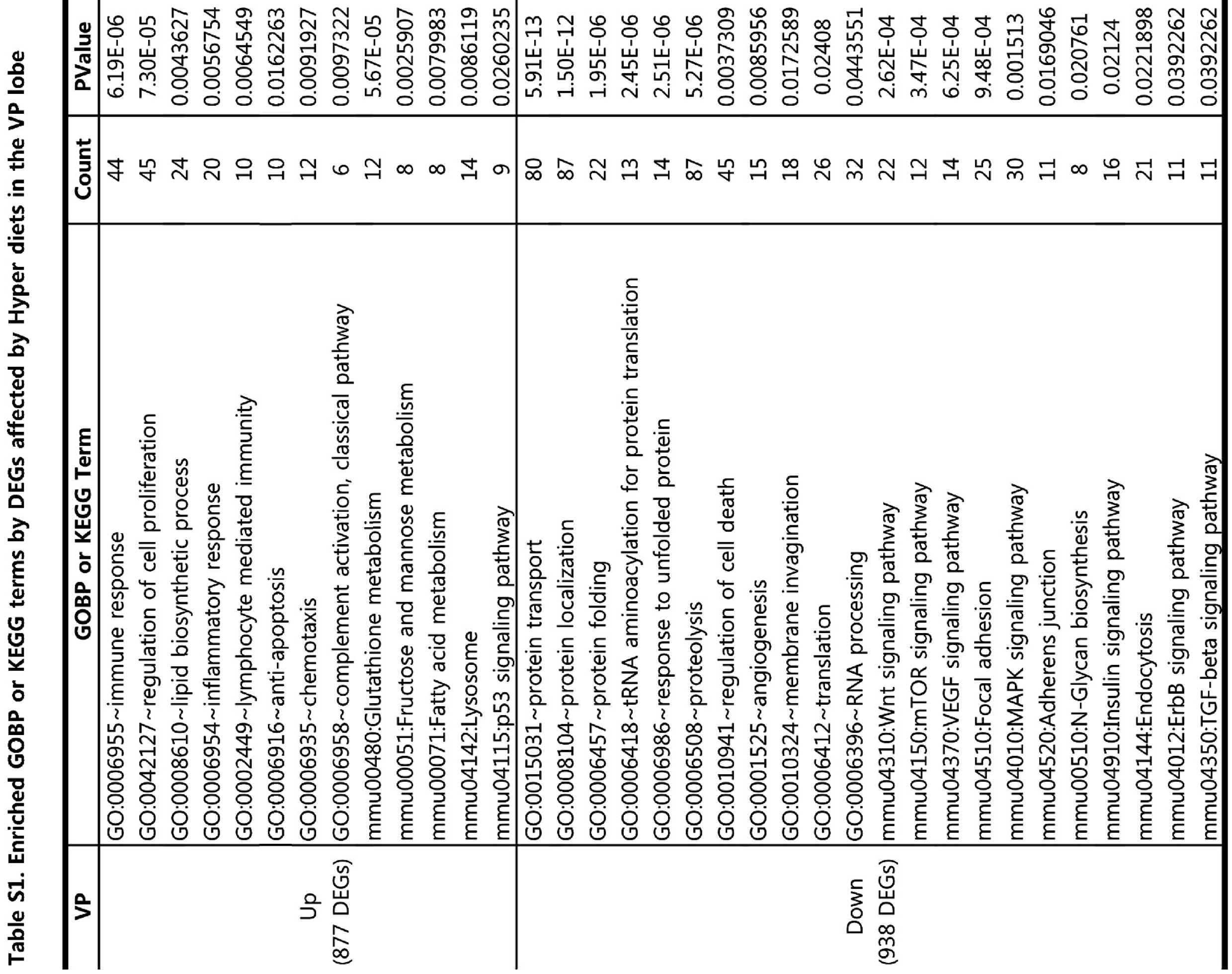

Supplement: Table S1 — Enriched GOBP or KEGG terms by DEGs affected by Hyper diet in the SCID VP lobe. (TIF) [file pone.0039448.s004.tif]

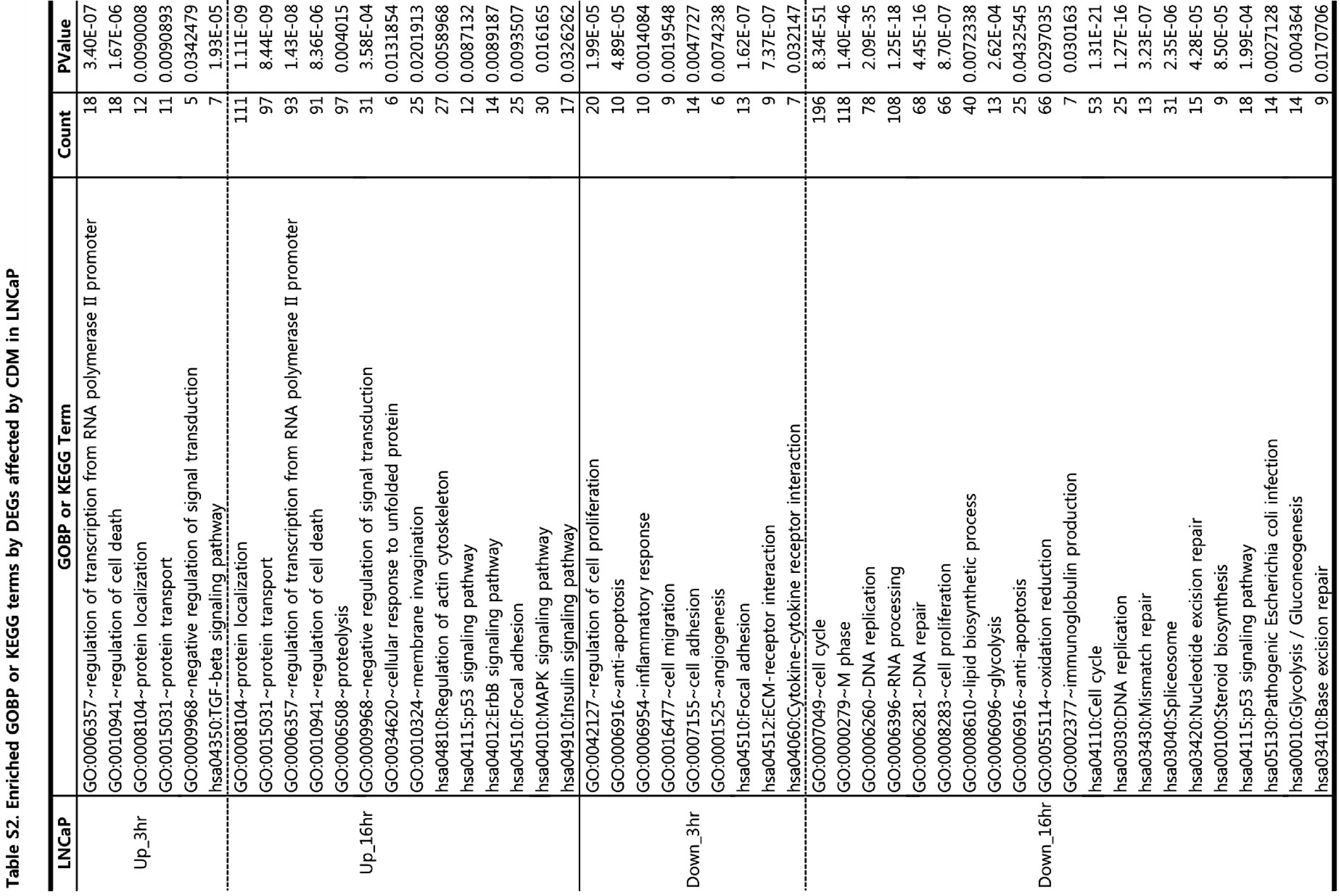

Supplement: Table S2 — Enriched GOBP or KEGG terms by DEGs affected by CDM in LNCaP cells. (TIF) [file pone.0039448.s005.tif]
